# CIPK9 is involved in seed oil regulation in *Brassica napus* L. and *Arabidopsis thaliana* (L.) Heynh.

**DOI:** 10.1186/s13068-018-1122-z

**Published:** 2018-05-02

**Authors:** Yanli Guo, Yi Huang, Jie Gao, Yuanyuan Pu, Nan Wang, Wenyun Shen, Jing Wen, Bin Yi, Chaozhi Ma, Jinxing Tu, Tingdong Fu, Jitao Zou, Jinxiong Shen

**Affiliations:** 1 National Key Laboratory of Crop Genetic Improvement/National Engineering Research Center of Rapeseed, Huazhong Agricultural University, Wuhan, 430070 China; 2 National Research Council Canada, Saskatoon, SK S7N0 W9 Canada; 3 Crop Research Institute of TIANJIN Academy of Agricultural Sciences, Tianjin, 300384 China

**Keywords:** *Brassica napus* L., Seed oil content, *CIPK9*, Sucrose, Seedling establishment, *Arabidopsis thaliana*

## Abstract

**Background:**

Accumulation of storage compounds during seed development plays an important role in the life cycle of oilseed plants; these compounds provide carbon and energy resources to support the establishment of seedlings.

**Results:**

In this study, we show that *BnCIPK9* has a broad expression pattern in *Brassica napus* L. tissues and that wounding stress strongly induces its expression. The overexpression of *BnCIPK9* during seed development reduced oil synthesis in transgenic *B. napus* compared to that observed in wild-type (WT) plants. Functional analysis revealed that seed oil content (OC) of complementation lines was similar to that of WT plants, whereas OC in *Arabidopsis thaliana* (L.) Heynh. *Atcipk9* knockout mutants (*cipk9*) was higher than that of WT plants. Seedling of *cipk9* mutants failed to establish roots on a sugar-free medium, but root establishment could be rescued by supplementation of sucrose or glucose. The phenotype of complementation transgenic lines was similar to that of WT plants when grown on sugar-free medium. Mutants, *cipk9*, *cbl2*, and *cbl3* presented similar phenotypes, suggesting that CIPK9, CBL2, and CBL3 might work together and play similar roles in root establishment under sugar-free condition.

**Conclusion:**

This study showed that *BnCIPK9* and *AtCIPK9* encode a protein kinase that is involved in sugar-related response and plays important roles in the regulation of energy reserves. Our results suggest that *AtCIPK9* negatively regulates lipid accumulation and has a significant effect on early seedling establishment in *A. thaliana*. The functional characterization of *CIPK9* provides insights into the regulation of OC, and might be used for improving OC in *B. napus*. We believe that our study makes a significant contribution to the literature because it provides information on how CIPKs coordinate stress regulation and energy signaling.

**Electronic supplementary material:**

The online version of this article (10.1186/s13068-018-1122-z) contains supplementary material, which is available to authorized users.

## Background

Sucrose is transported from leaves to other tissues as a main product of photosynthesis, and it is also the main carbon and energy source for plants’ reproduction and growth and for obtaining storage components such as oil, starch, and protein [1–4]. In oilseeds, lipids are the major energy reserves and are stored in the form of triacylglycerols (TAGs) in oil bodies [5, 6]. Once germination begins, consumption of the energy reserves accumulated during seed maturation is necessary for energy production to ensure heterotrophic growth [7–10]. During the early postgermination stage, lipases initiate the hydrolysis of TAGs into glycerol and fatty acids (FAs), and the β-oxidation pathway depredates those FAs for carbon [11–16]. During seed germination, the young plant degrades energy reserves and transfers them into soluble molecules (e.g. sucrose), which can be transported throughout the plant.

As a main carbon and energy source, sugars function as signaling molecules, represent the nutrient status of the plant, and regulate many nutrient-related genes [17–19]. In addition, sugars can regulate many biological processes, including starch synthesis, cell division, and growth. Conversely, sugar starvation can affect the plant’s central development by enhancing photosynthetic activities and carbon remobilization [20, 21]. Sugar signaling can interact with several other signals including hormone, nitrogen [22], stress, and energy levels [23–25]. However, there are few transcriptional factors (TFs) among the large number of genes regulated by sugars. A novel screening technology demonstrated the participation of several basic region–leucine zipper (bZIP) and v-myb avain myeloblastosis viral oncogene homolog (MYB) TFs in the sugar signaling system [18, 26–29]. The *cis*-acting elements found among the target genes provide clues for identifying *trans*-acting factors in sugar response. For instance, the protein kinase SnRK1A has been identified as a sugar response transcriptional factor (TF) in rice, based on the *cis*-acting elements present in the promoter of gene α-amylase 3 (α-*Amy3*) [30]. Acting both as a structural component and as an energy source, sugar is an important substrate for plants during their active growth, seed production, and response to stress.

Energy-signaling protein kinases are conserved among the different species of eukaryotes: SNF1 in yeast, AMP-activated protein kinase (AMPK) in mammals [31–35], and SnRK1 [36–40], and SnRK2.6 in plants [41]. The Snf1-related protein kinases (SnRKs) found in plants comprise families SnRK1, SnRK2, and SnRK3, which include three, 10, and 25 members, respectively [40, 42]. Given their ability to interact with calcineurin B-like proteins (CBLs) [43–50], members of the SnRK3 family are also named CBL-interacting protein kinases (CIPKs). However, their roles in energy signaling and stress response remain unknown. To unravel the roles of the CIPKs in the regulation of carbohydrate and energy metabolism, we aimed to identify the kinases involved in seed oil production. In oilseed plants, such as rapeseed, a large proportion of photoassimilate is transported to seeds for TAG synthesis, which demands abundant energy and carbon sources. The carbon level relative to that of nitrogen is then used as a signal to accelerate or decelerate the rate of oil synthesis [51, 52]. Overall, the analyses performed in these previous studies suggested that sugar and energy supplies in source tissues of oilseed plants affect seed oil synthesis.

Our reverse genetic study suggested that *BnCIPK9* is a negative regulator of seed oil synthesis in *B. napus*. To further elucidate its function, we overexpressed *BnCIPK9* in *B. napus*, and demonstrated its roles in reducing OC. In the present study, we also investigated the function of *Arabidopsis thaliana* (L.) Heynh. *AtCIPK9* in lipid accumulation and its role in root establishment.

## Results

### Phenotypic variation

**Fig. 1:**
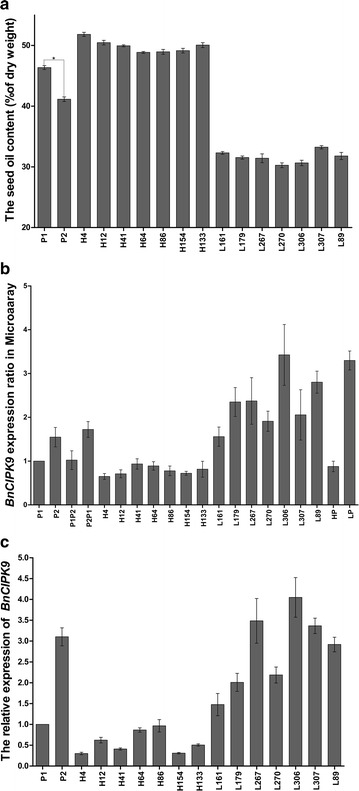
Negative regulation of *BnCIPK9* according to seed oil content in *Brassica napus*. **a** Seed oil contents of various rapeseed lines expressed as a percentage of dry weight. For P1 and P2, Bars represent mean values of seed oil content, and error bars indicate its standard deviation (SD) (*n* = 2–4); the asterisk indicates a significant difference (*P* < 0.05) in seed oil contents between P1 and P2. **b** Different expression levels of *BnCIPK9* in the high-oil and low-oil content lines, as determined by microarray analysis. Error bars indicate SD (*n* = 6). **c** Relative expression analysis of *BnCIPK9* in the rapeseed lines examined. The data are mean ± SD of three replicates

Seeds from the parental lines, P1 (high-oil parent) and P2 (low-oil parent), showed a statistically significant difference in their seed oil content (OC): 46.4% (± 0.7; *n* = 4) and 41.1% (± 0.9; *n* = 2), respectively (Fig. 1a). The OC exhibited transgressive segregation, with a minimum of 30.3% (± 0.8) and a maximum of 51.8% (± 0.7) in the F_2_ populations of P1/P2 (Fig. 1a). Within these populations, the seven lines with the highest OC were H4 (51.8%), H12 (50.5%), H133 (50.1%), H41 (49.9%), H154 (49.1%), H86 (49.0%), and H64 (48.9%), and the seven lines with the lowest OC were L307 (33.2%), L161 (32.3%), L89 (31.8%), L179 (31.5%), L267 (31.4%), L306 (30.7%), and L270 (30.3%).

Microarray analysis conducted using the above-mentioned rapeseed lines allowed identifying 10 genes potentially involved in OC regulation, including *BnCIPK9*. Gene expression analysis showed that *BnCIPK9* was differentially expressed between the high- and the low-oil content lines from F_2_ populations, with higher expression in the low-oil lines than in the high-oil content lines (Fig. 1b). The results of quantitative real-time PCR (qRT-PCR) analysis further confirmed a negative correlation between the expression level of *BnCIPK9* and the OC of rapeseed seeds (Fig. 1c).

The YlSNF1 is known to negatively regulate lipid accumulation in yeast [53]. Consistent with this finding, our results suggested that *BnCIPK9* was a negative regulator of OC in rapeseed. Therefore, we further attempted to characterize the molecular mechanism underlying this regulation. Full-length *BnCIPK9* sequences, including the 5′-untranslated region (5′-UTR) were obtained from rapeseed lines. Analysis of rapeseed genome suggested that *BnCIPK9* has four copies, and that is conserved (data not shown). Annotation of the 3050-bp (*BnCIPK9* promoter1) and a 3372-bp (*BnCIPK9* promoter2) fragments isolated from the 5′-UTR next to the *BnCIPK9* genomic locus from the parental line P1 revealed several *cis*-elements in this region. The main difference between *BnCIPK9* promoter1 and promoter2 sequences was the presence of a 264 bp insertion/deletion at the position − 281 in *BnCIPK9* promoter2 (Fig. 4a).

### Expression analysis of *BnCIPK9*

**Fig. 2:**
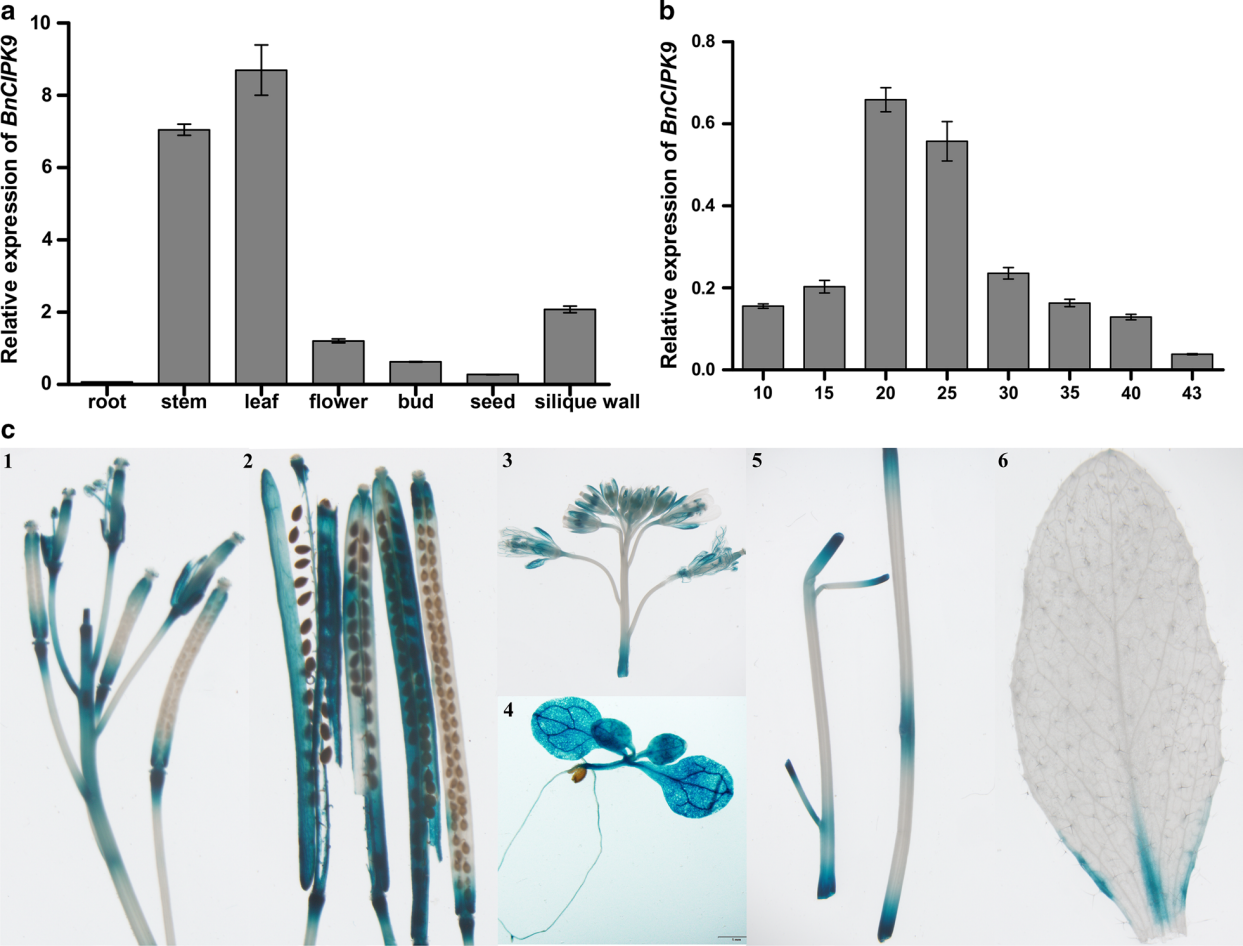
Expression patterns of *BnCIPK9* in the high-oil content parent (P1). **a** Spatial and temporal expression analyses of *BnCIPK9* in roots, stem, leaves, 24 days after pollination (DAP) seeds, 24DAP slique wall, buds, and flowers using quantitative real-time PCR (qRT-PCR). Total RNA was isolated from the different tissues (roots, stem, leaves, 24DAP seeds, 24DAP silique wall, buds, and flowers); qRT-PCR was performed with *BnCIPK9*-specific primers and *BnaUBC9*-specific primers. Gene *BnaUBC9* was used as an internal control for normalization. The data shown are mean ± standard deviation (SD) of three technical replicates. **b** *BnCIPK9* expression profile using qRT-PCR at different seed-development stages (10, 10DAP; 15, 15DAP; 20, 20DAP; 25, 25DAP; 30, 30DAP; 35, 35DAP; 40, 40DAP; 43, 43DAP). Tissues were collected at different seed-development stages, and RNA was isolated to obtain first-strand cDNA. The qRT-PCR was performed with *BnCIPK9*-specific and *BnaUBC9*-specific primers. *BnaUBC9* expression levels were used as an internal control. The data shown are mean ± SD of three technical replicates. **c** GUS staining of different tissues in *BnCIPK9:GUS* transgenic plants. Gus activity in 7-day-old seedlings (4) and individual organs of adult plant (1–3, 5, 6), siliques (1, 2), whole inflorescence (3), stem (5), mature leaf (6). Scale = 2 mm (siliques, whole inflorescence, stem, mature leaf), 1 mm in 7-day-old seedlings

The *A. thaliana* homolog of *BnCIPK9*, *AtCIPK9*, is expressed in several tissues, including leaves, stem, flowers, and siliques [54–56]. At 24 days after pollination (DAP), high *BnCIPK9* transcription levels were observed in the stem, leaves, and in silique walls, whereas low transcription levels were found in flowers, buds, 24 DAP seeds, and especially in the roots (Fig. 2a). A qRT-PCT was performed to characterize the relative accumulation of mRNA transcripts of *BnCIPK9* during the different developmental stages of P1 seeds, from 10 to 43 DAP (Fig. 2b). Transcript levels were relatively low during early seed development (up to 15 DAP), but a sharp increase was observed from 15 to 20 DAP, after which transcript levels gradually decreased from 25 to 43 DAP (Fig. 2b). These results indicated that the expression pattern of *BnCIPK9* is similar to that of *AtCIPK9*; both are expressed in various organs, including photosynthetic and non-photosynthetic tissues.

The expression profiles of *BnCIPK9* were also evaluated based on *β*-glucuronidase (GUS) activity in transgenic *A. thaliana* seedlings carrying *GUS*, under the control of *BnCIPK9* promoters 1 and 2 (*BnCIPK9* promotor1:*GUS*, and *BnCIPK9* promotor2:GUS). This analysis revealed a similar expression pattern between plants transformed with *BnCIPK9* promotor1:GUS (Fig. 2c) and *BnCIPK9* promotor2:GUS (data not shown). Wounding stress signal strongly induced the expression of *BnCIPK9* (Fig. 2c2, c3, c5). Although *BnCIPK9* was expressed in siliques, GUS activity was mainly restricted to the stigma and receptacle of developing siliques (Fig. 2c1, c2). In the inflorescence, substantial GUS activity was predominantly detected in the anthers and stamen filaments and in the vasculature of mature petals and sepals (Fig. 2c1, c3). In addition, *BnCIPK9* was expressed in all tissues of 7-day-old seedlings, particularly in vascular tissue of leaves (Fig. 2c4). In adult plants, low GUS activity was detected in the typical rosette leaf and old stems (Fig. 2c5, c6). Overall, GUS activity profiles were generally consistent with the mRNA profiles obtained using qRT-PCR.

### Decrease in lipid storage in the seeds of transgenic plants overexpressing *BnCIPK9*

**Fig. 3:**
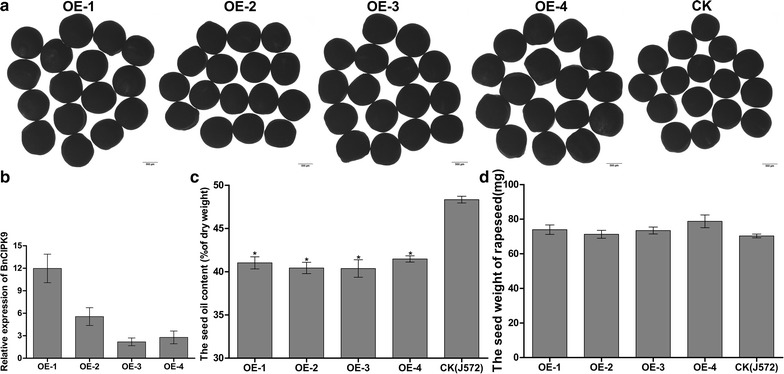
Transgenic rapeseed plants overexpressing *ProBnNapin*:BnCIPK9T178D (*BnCIPK9*-OE) and analyses of seed oil content, seed size, and seed weight. **a** T3 generation of dried seeds of four independent ProBnNapin:BnCIPK9T178D overexpressing transgenic lines (OE-1, OE-2, OE-3, OE-4) and J572 (wild type, WT), used as the control. Bars correspond to 500 µm. **b** Expression levels of *BnCIPK9* in the seeds of transgenic rapeseed lines as assessed by qRT-PCR. Bars represent mean ± standard deviation (SD) (three technical replicates). **c** Total seed oil contents of transgenic rapeseed plants (T2), and J572 (WT) used as the control. Bars indicate the SDs of different replicates. Asterisks indicate significant differences (*P* < 0.05) between transgenic and control (J572) plants. **d** Dry seed weight (15 seeds) of transgenic and J572 rapeseed plants. Error bars indicate the SD (*n* ≥ 5)

We generated transgenic rapeseed plants overexpressing *BnCIPK9* to determine whether an increase in *BnCIPK9* expression would reduce the OC in seeds. To specifically control for the accumulation of lipids in mature seeds, the *BnCIPK9* transgene was expressed under the control of seed-specific *BnNapin* promoter. We recovered four independent lines the pattern and rate of growth, leaf number, and leaf size of which were all normal. The T3 mature seeds from the transgenic plants were normal in size (Fig. 3a, d), and transgenic plants had high levels of *BnCIPK9* expression (Fig. 3b). Seeds harvested from four fully mature transgenic plants were used for further studies. The transgenic plants with the increased levels of endogenous *BnCIPK9* expression had a significantly lower OC than the non-transgenic plants (Fig. 3c).

### Characterization of promoters 1 and 2 of *BnCIPK9*

**Fig. 4:**
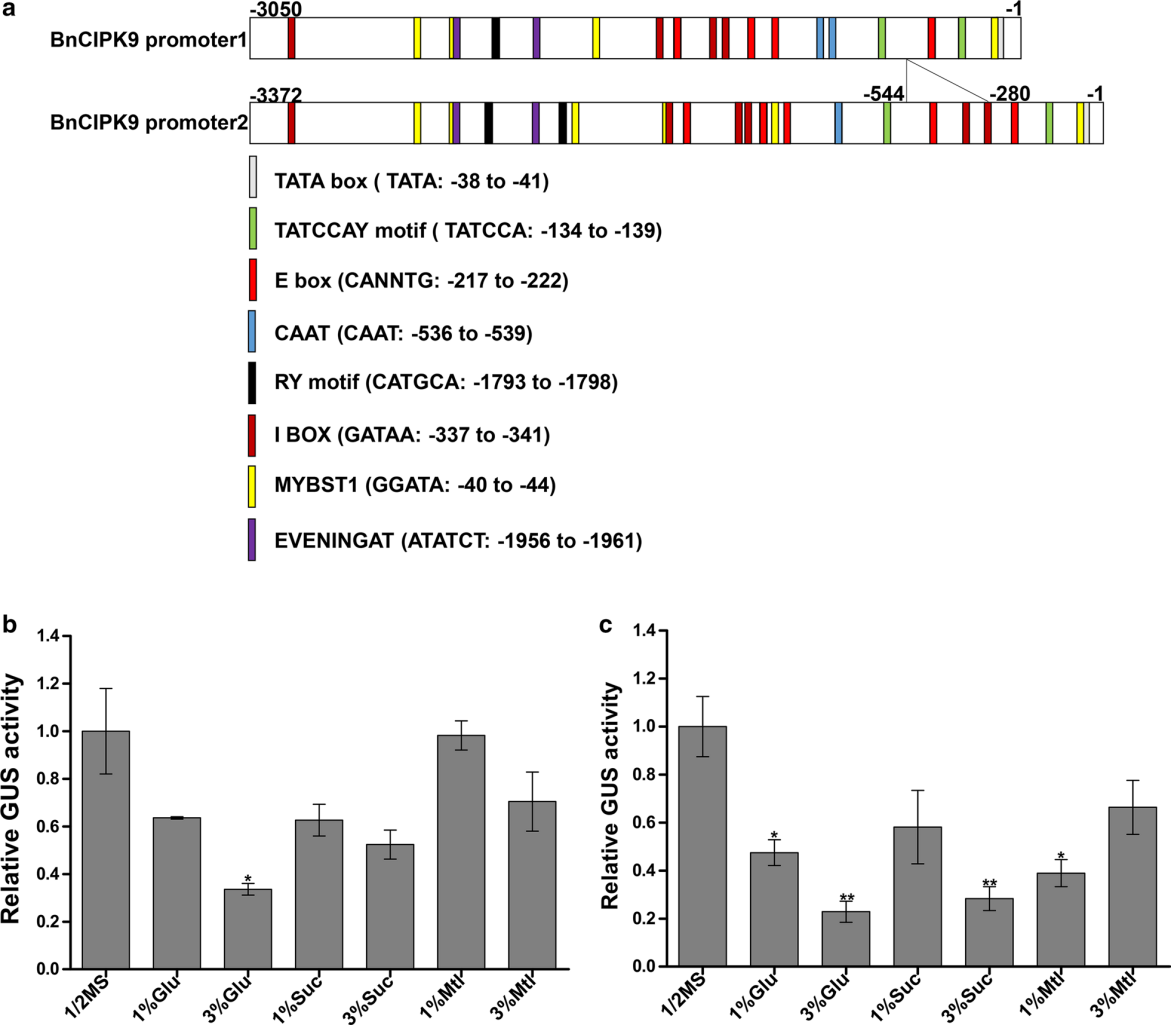
Promoter and expression pattern of *BnCIPk9* in seedlings, in the presence and absence of sugars. **a** Composition of putative *cis*-acting elements in the *BnCIPK9* promoter *(BnCIPK9* promoter1, 3050 bp from the original fragment; *BnCIPK9* promoter2, 3372 bp from the original fragment). **b** Activity of *BnCIPK9* promoter1 in 7-day-old seedlings in the presence and absence of sugars. The transgenic fusion including 3050 bp (*BnCIPK9* promoter1) of the 5′-upstream regulatory region of the *BnCIPK9*, fused to the GUS reporter gene used to generate the transgenic lines (*BnCIPK9* promoter1:GUS). **c** Activity of the promoter2 of *BnCIPK9* in 7-day-old seedlings in the presence or absence of sugars. The transgenic fusion included 3372 bp (*BnCIPK9* promoter2) of the 5′-upstream regulatory region of *BnCIPK9*, fused to the GUS reporter gene used to generate the transgenic lines (*BnCIPK9* promoter2:GUS). Different sugar sources [1 or 3% glucose (Glu), or sucrose (Suc)] were used; mannitol (Mlt) was used as a control for osmotic stress. Data are shown as means of the relative GUS activity of promoter1 and promoter2 of *BnCIPK9*± standard deviation (SD) (*n* = 3). The Student’s *t* test was performed to evaluate the significance of differences between means at *P < 0.05; **P < 0.01

To determine the regulatory elements of *BnCIPK9*, the promoter regions (3.0 kb fragment upstream of the translation start site) were isolated from the rapeseed genomic DNA. We performed an in silico analysis of *BnCIPK9* promoter1 (3050 bp) and *BnCIPK9* promoter2 (3372 bp) fragments to find the *cis*-elements related with sugar response. The analysis of the promoter fragments was performed using the PLACE database (http://www.dna.affrc.go.jp/PLACE), searching for the motifs that might be involved in gene suppression by sugars (Fig. 4a). Seventeen and 23 potential sugar response motifs were characterized from *BnCIPK9* promoter1 and promoter2, respectively. One of the most interesting elements found in the promoters was the TATCCA motif, which has also been found in the 5′-UTR of *α*-*Amy3D* from rice and characterized as a sugar response motif [57, 58]. This motif occurs twice in promoter1 (at − 134 and − 391 from ATG) and promoter2 (at − 134 and − 661 from ATG), separated by 252 and 516 bp, respectively. The I-box [59] was found four times within *BnCIPK9* promoter1 and six times within *BnCIPK9* promoter2, but only one I-box motif of *BnCIPK9* promoter2 overlapped with the MYBST1 element [60] (Fig. 4a). The MYBST1 element occurred four times within *BnCIPK9* promoter1 and six times within *BnCIPK9* promoter2. The E-box motifs [61, 62], which are likely seed-specific, occurred four times within *BnCIPK9* promoter1 and five times within *BnCIPK9* promoter2.

To characterize the molecular mechanism of the regulation of *BnCIPK9* by sugars, fragments of *BnCIPK9* promoter1 and *BnCIPK9* promoter2 upstream the ATG codon were used for GUS analysis. We examined the effects of sucrose and glucose (1 or 3% w/v) supplementation in half-strength Murashige and Skoog (MS) medium on GUS expression. As a control, mannitol was used for imposing osmotic stress. Supplementation of 3% glucose significantly reduced GUS expression in promoter1:GUS lines compared to the medium without supplementation (Fig. 4b). Both 1% glucose and 1% mannitol resulted in lower GUS activity for the *BnCIPK9* promoter2 in relation to the sugar-free condition (Fig. 4c). In the presence of both 3% sucrose and 3% glucose, a significant reduction in the GUS activity was observed in *BnCIPK9* promoter2:GUS transgenic lines, compared that observed on the medium without supplementation (Fig. 4c). Quantitative GUS assays suggested that the expression of *BnCIPK9* promoter1 and *BnCIPK9* promoter2 was reduced at different extents in the presence of sugar, and that these regions were particularly responsive to sucrose and glucose.

### Characterization of the promoter region of *AtCIPK9*

**Fig. 5:**
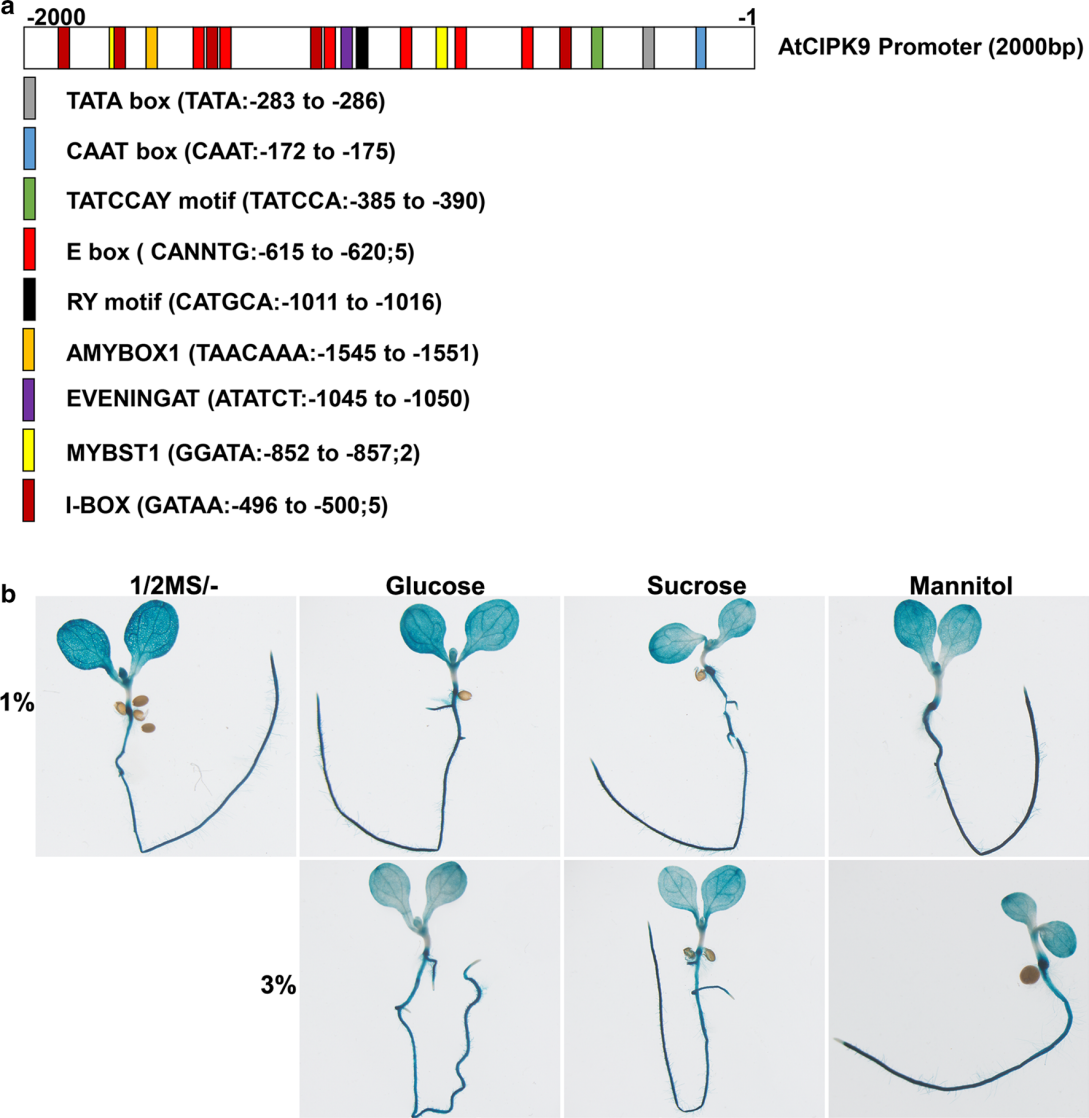
Promoter and expression pattern of *AtCIPK9* in 7-day-old seedlings in the presence or absence of sugars. **a** Composition of putative *cis*-acting elements in promoter of *AtCIPK9*. The promoter of *AtCIPK9* is the 2000 bp from the original fragment. **b** Expression pattern of the *AtCIPK9* in 7-day-old seedlings in the presence or absence of sugars. The transcriptional fusion included 3.0 kb of the 5′-untranslated region of the *AtCIPK9* fused to the GUS reporter gene used to generate the transgenic lines (*ProAtCIPK9*:GUS). Different sugar sources (1 or 3% glucose, or sucrose) were used: mannitol was used as a control for osmotic stress. The images shown are representative of the three biologically independent experiments

The promoter region of *AtCIPK9* contains two putative sugar-responsive elements, TATCCA and TAACAAA [63], which are found in gene *α*-*Amy3*. In addition, the promoter fragment includes an RY and an EVENINGAT motif [64, 65]. Five I-box motifs were found in the promoter of *AtCIPK9*, but only one I-box motif overlapped the MYBST1A motif. The promoter of *AtCIPK9* includes six E-box motifs, which potentially mediate gene expression in seeds. On half-strength MS supplemented with 1% sucrose, a reduction in the GUS activity was observed in *AtCIPK9* promoter:GUS transgenic lines compared to that observed on the medium without sugar supplementation (Fig. 5b). On half-strength MS with 1% glucose, GUS expression in *AtCIPK9* promoter:GUS transgenic lines was indistinguishable from that observed in the absence of sugar. In the presence of both 3% sucrose and 3% glucose, GUS activities were reduced observed in the *AtCIPK9* promoter:GUS transgenic lines compared to that observed on the medium without sugar supplementation.

### Disruption of *AtCIPK9* leads to an increase in TAG in seeds and in the failure establishment of root

**Fig. 6:**
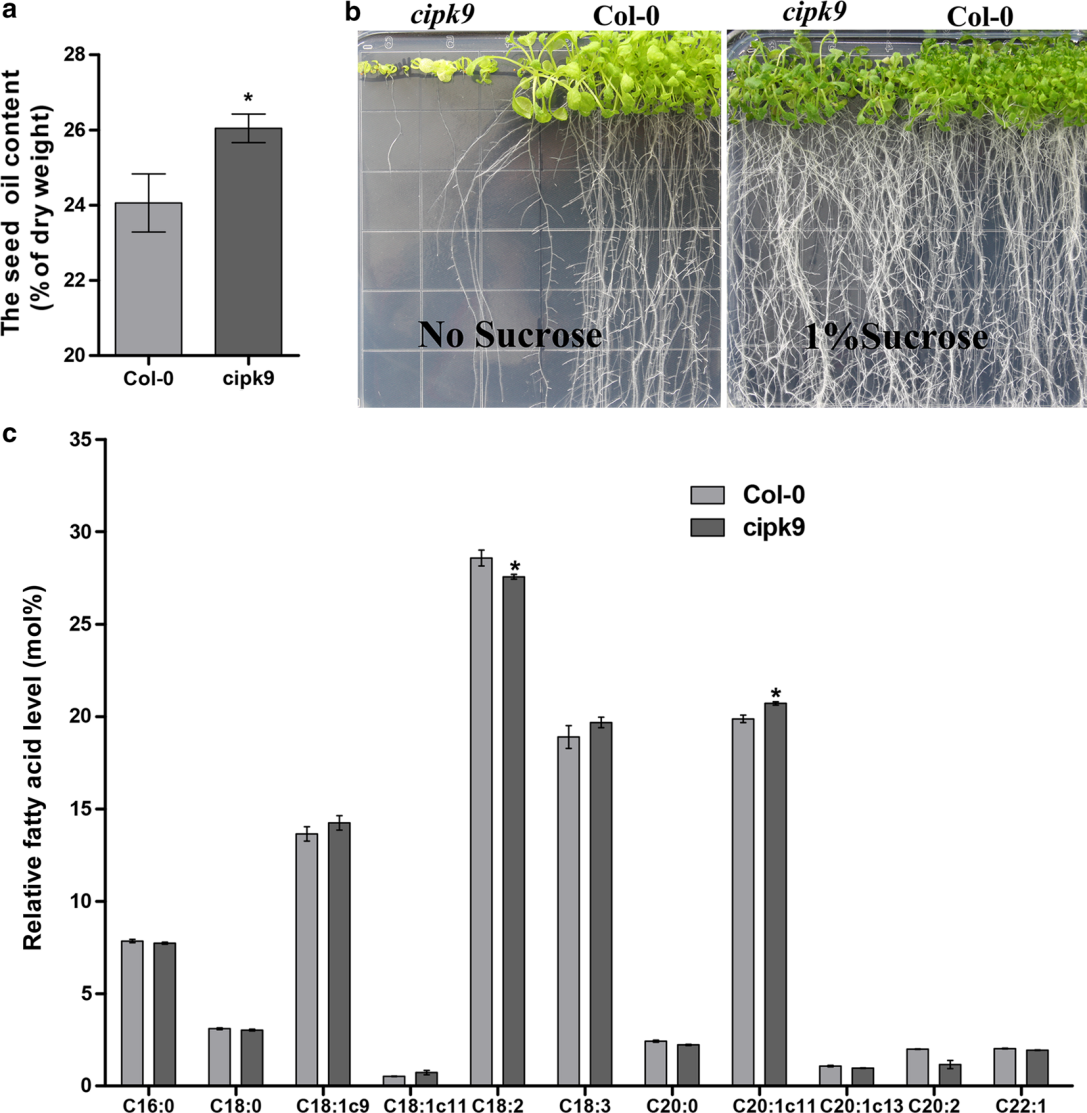
Triacylglycerol levels and seedling establishment in the *cipk9* mutant. **a** Relative fatty acids (FAs) in dried Col-*0* and *cipk9* seeds. **b** Hypersensitive growth of *cipk9* mutants in media with no sugar added. **c** FA profile in Col-*0* and *cipk9* seeds. The student’s *t* test was performed to evaluate the significance of differences between means at *P < 0.05. Error bars correspond to standard deviations (*n* ≥ 5)

We obtained an *A. thaliana* mutant (Salk_058629) from the ABRC Stock Center containing a transfer-DNA insertion in the fourth exon (1115 bp from ATG) of the *AtCIPK9* (At1g01140) locus [54]. This mutant was designated *cipk9*. The transcript level of *AtCIPK9* in *cipk9* was 40-fold lower than that in wile-type (WT) plants during the early stage of silique development (6–10 DAP). Previous results from northern blot analysis also showed that the transcription of *AtCIPK9* in this mutant line was disrupted [55]. The average OC in WT plants was 24.1%, whereas in *cipk9* mutant plants it was 26.3% (Fig. 6a). In *cipk9* seeds, the relative proportion of C20:1∆11 was increased, whereas the relative proportion of C18:2 was clearly decreased, compared to WT plants (Fig. 6c).

**Fig. 7:**
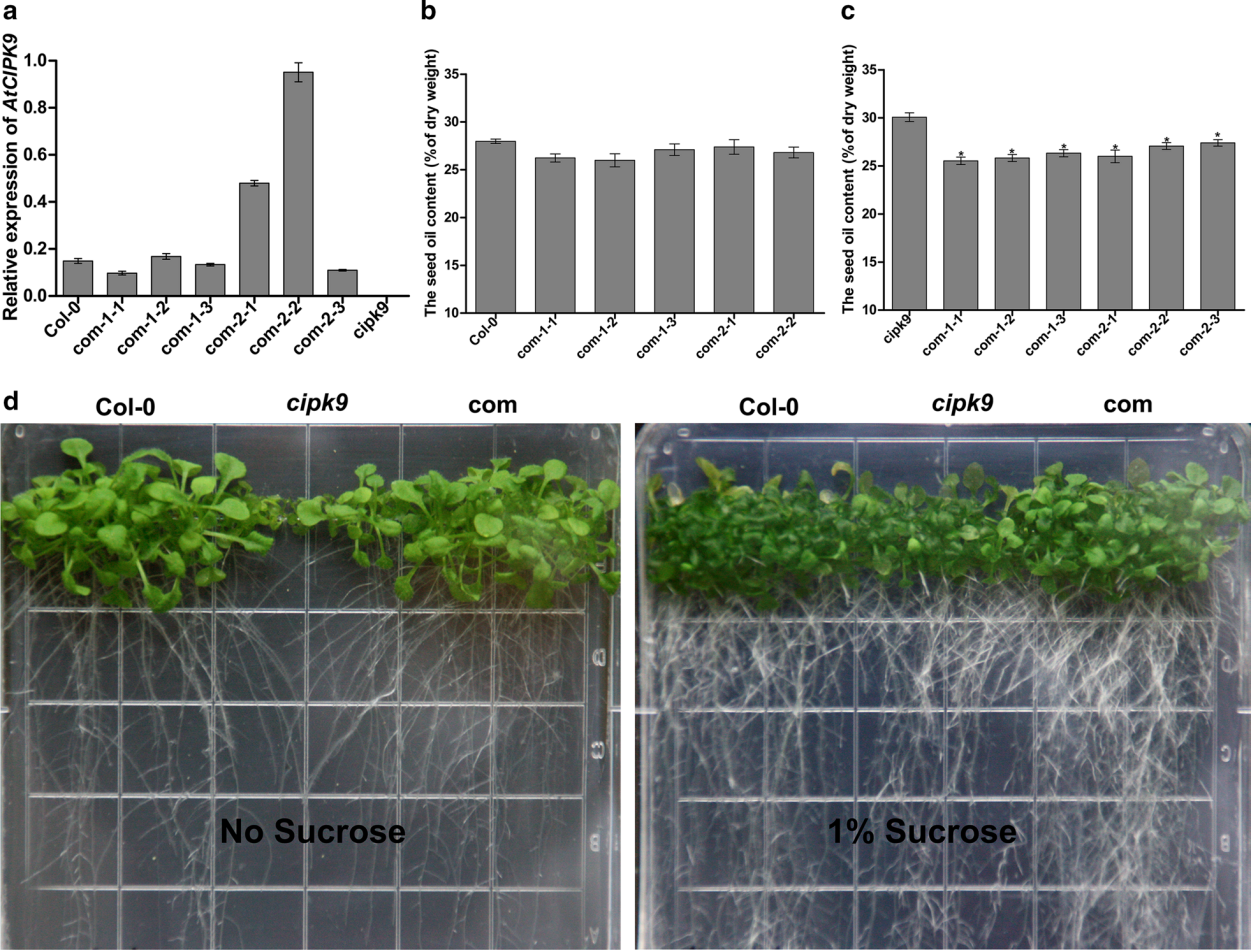
Complementation of *cipk9* mutant and analysis of seed oil content. **a** *AtCIPK9* transcript levels in the transgenic lines. **b** Seed oil contents of T3 transgenic and wild type (WT, Col-0). **c** Seed oil contents of T3 transgenic and *cipk9* mutants. **d** Phenotypes of the complementation lines of *cipk9*, WT (Col-0), and *cipk*9 grown in half-strength Murashige and Skoog medium without sucrose for 7 days. The student’s *t* test was performed to determine the significance of differences between means at *P < 0.05. Error bars indicate standard deviations (*n* ≥ 5); com-1-1, com-1-2, and com-1-3 are three independent promoter*AtCIPK9*:*AtCIPK9* (genomic DNA) transgenic lines; com-2-1, com-2-2, com-2-3 are three independent promoter*AtCIPK9*:*AtCIPK9* (coding sequence) transgenic lines

To examine defects in seedling establishment on half-strength MS medium, we examined seedlings’ generation and establishment in the absence and presence of 1% sucrose. No significant differences were found between the *cipk9* mutant and WT plants under normal growth conditions (half-strength MS medium with 1% sucrose), although *cipk9* plants were more sensitive to the half-strength MS medium without sugar supplementation, as they failed to establish roots under this condition. To demonstrate the genetic complementation of *AtCIPK9*, we transformed mutant plants using the genomic DNA (com-1) and the coding sequence (com-2) of *AtCIPK9* under the control of its native promoter. Three independent complementation lines were generated for the allele of *cipk9*. The expression levels of *AtCIPK9* were restored in the three complementation lines (Fig. 7a), and the phenotypes of these three complementation lines were similar to that of WT plants, when grown on half-strength MS medium without sugar supplementation (Fig. 7d). The three transgenic lines showed normal OC (Fig. 7b), although significantly lower than that of *cipk9* mutant plants (Fig. 7c), implying that *AtCIPK9* is required for proper OC. These results demonstrated that sugar sensitivity and lipid accumulation in seeds of *cipk9* were due to the disruption of *AtCIPK9*. Plants appeared normal on the medium lacking sucrose, suggesting that *AtCIPK9* is required for proper early seedling establishment.

### CBL2 and CBL3 are involved in the upstream regulation of CIPK9

**Fig. 8:**
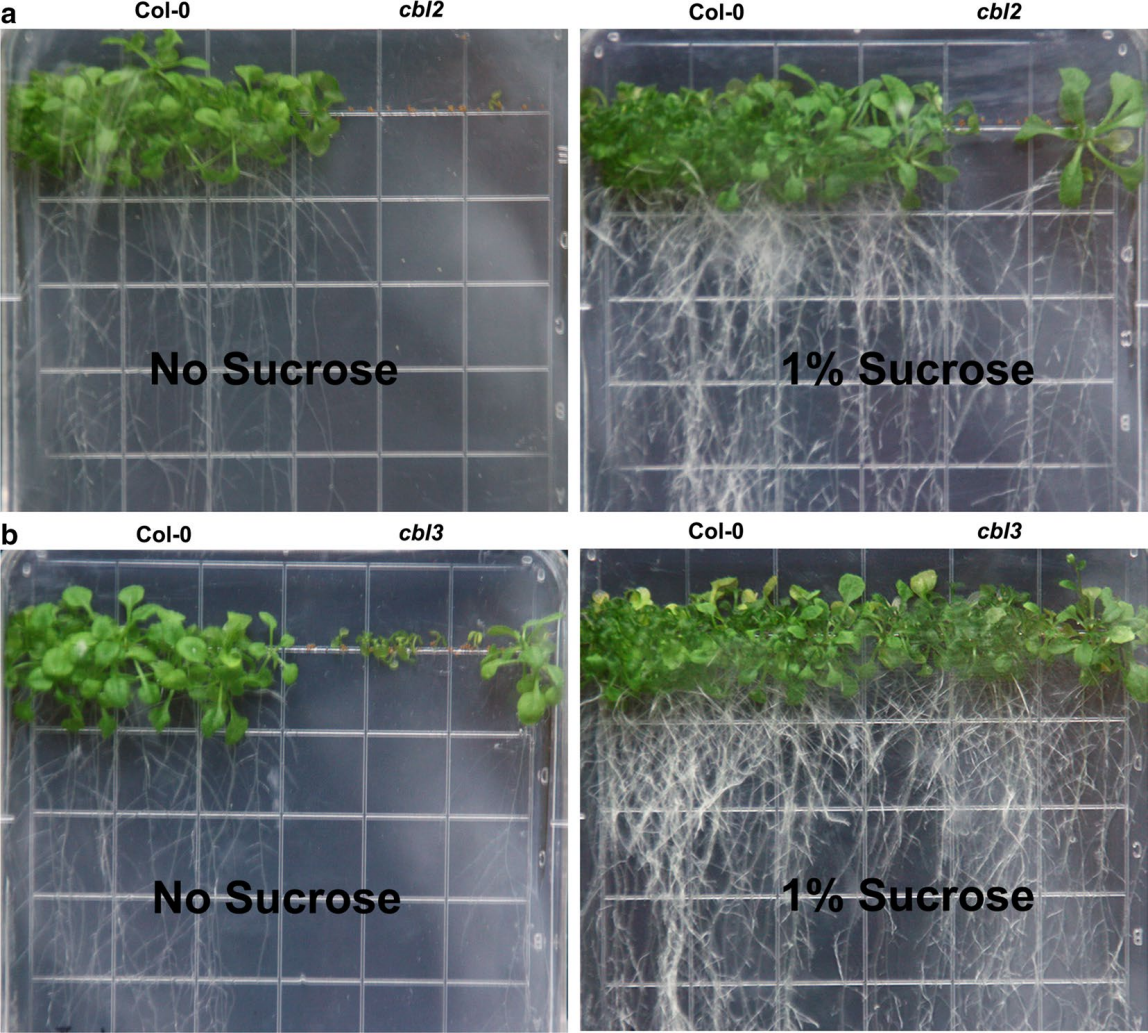
Postgermination growths of *clb2* and *clb3*. **a** Phenotype tests performed to *cbl2* mutants grown in half-strength Murashige and Skoog (MS) medium without sucrose for 7 days. **b** Phenotype tests performed to *cbl3* mutants grown in half-strength MS medium without sucrose for 7 days

It has been shown that CBL2 and CBL3 interact with CIPK9 [56]. We o<btained *cbl2* (SALK_057048C) and *cbl3* (SALK_091827C) mutants from the ABRC Stock Center, and the expressions of CBL2 and CBL3 were, respectively, disrupted in *cbl2* and *cbl3* mutants [56, 66]. Both mutants showed the typical *cipk9* phenotype on the half-strength MS medium without sugar (Fig. 8a, b), suggesting that CBL2, CBL3, and CIPK9 might work together and play roles in the establishment of seedlings in the absence of sugar.

## Discussion

In eukaryotes, the regulators involved in lipid metabolism and carbohydrate storage in the SNF1/AMPK family of protein kinases are evolutionarily conserved from yeast (SNF1) to mammals (AMPK) and plants (SnRKs). Similar to a previous study that demonstrated the negative regulation of lipid accumulation by SNF1 in yeast [53], we showed that BnCIPK9 and AtCPK9 are negative regulators of OC, and that CIPK9 might also regulate OC by influencing the expression of genes related to lipid metabolism. However, SnRK2.6 is a positive regulator of seed oil production in *A. thaliana* [41]. Considering that YlSnf1 apparently regulates lipid metabolism at the transcriptional level, it might also regulate lipid metabolism by posttranscriptional regulatory processes, including SNF1-dependent translocation and phosphorylation of the key enzymes. When active, Snf1 inhibits the acetyl-CoA carboxylase (ACCase) [67, 68], and glycerol phosphate acyltransferase (GAPT) [69] activities. In fact, the first conserved function shown for SNF1/AMPK protein kinase among eukaryotes was the regulation of ACCase, the first and rate-limiting enzyme in the de novo synthesis of FAs [67, 68, 70–72]. In fact, ACCase is highly regulated through both transcriptional and biochemical mechanisms, and it is critical for controlling the influx of carbon into FAs, and thus into oil biosynthesis [73–79]. Nutritional and metabolic signals, such as glucose limitation and salt stress, are transduced to ACC1 by AMPK [32, 35, 80]. In plants, SnRK1 negatively regulates 3-hydroxy-3-methyl-glutaryl-coenzyme A reductase [81], and is supposed to negatively regulate diacylglycerol acyltransferase (DGAT) [82]. A putative tyrosine phosphorylation motif was observed in mammalian DGAT, but no apparent tyrosine phosphorylation site could be found in transient axonal glycoprotein1 [83]. However, visual examination revealed a consensus sequence (X-L200-X-K202-X-X-S205-X-X-X-V209), which was identified as the targeting motif, typical of the members of the SnRK1 protein kinase family [84]. Similar to SnRK1 in plants and Snf1 in yeast, CIPK9 might also regulate the activities of these enzymes through protein phosphorylation.

In oilseed plants, an essential function of seed reserves, manifested by OC levels, is to provide energy for postgermination growth until the seedling can perform photosynthesis [7, 85]. We observed a higher OC in *cipk9* mutants than in WT plants, but the roots of *cipk9* mutant seedlings failed to establish on half- strength MS medium without sugar, which could be recovered by the addition of exogenous sucrose. Thus, *cipk9* resembles the *sdp1* mutant, which showed increased OC because of a defect in TAG degradation [13, 86, 87]. During the establishment of seedling roots, resources are mobilized by hydrolysis of lipids and fatty acid catabolism (*β*-oxidation), which connected to sugar biosynthesis via the glyoxylate cycle [15]. It has been shown that seedling establishment is compromised in plants with deficient glyoxylate cycle [88, 89], gluconeogenesis [90, 91], and transportation of intermediates derived from lipid breakdown [92–96]. The establishment of seedlings could be rescued by the supplementing sucrose to mutants with deficient seed oil catabolism [91], but carbohydrate metabolism [97] was affected during seed development. Furthermore, FAs and FA-derived lipids can facilitate successful seed germination and seedling establishment [6].

Glycolysis, tricarboxylic acid cycle, oxidative phosphorylation, and mitochondrial electron transport were reported to be significantly upregulated in germinating seeds of *A. thaliana*, indicating that respiration is one of the essential processes to facilitating seed germination [98, 99]. The *cipk9* mutants grown in the medium supplemented with sugar showed similar root length to WT plants, indicating that plants are capable of sucrose catabolism. Transcript analysis indicated that *AtCIPK9* has high expression in root, flower, developing silique, and young seedlings [54–56]. Glycolysis and gluconeogenesis are important biological process for providing energy and structural components, which is critical for seedling establishment. These results clearly show that *AtCIPK9* plays an important role during germination and the later phase of seedling establishment. Thus, *cipk9* seeds might not be able to fully convert lipids to sucrose.

The expression of *BnCIPK9* and *AtCIPK9* are induced by sugar starvation and suppressed by sugar supplementation, which is similar to the regulation pattern of *DIN6*, *STP1*, and α-*Amy3* [24, 58, 100, 101]. In the present research, we attempted to identify the *cis*-acting elements required for the suppression of *BnCIPK9* by sugar. In silico assays showed that *BnCIPK9* and α-*Amy3*, *STP1*, and *DIN6* promoters shared two TATCCA *cis*-acting elements and G boxes [24, 58, 100]. The TATCCA *cis*-element was identified as the binding site for OsMYB2, and is essential for the regulation of *α*-*Amy3* in rice by sugar [68]. Moreover, the arrangement of these elements in *BnCIPK9* (in tandem and separated by 252 bp and 516 bp) is different from that in α-Amy3 (separated 15 bp) [57]. As for *AtCIPK9*, the promoter *JcSDP1* also carries one TATCCA element; gene expression is sugar-dependent and it is especially responsive to sucrose and fructose [95]. Thus, the TATCCA element is an interesting candidate for better understanding the mechanism by which sugar regulates *BnCIPK9*. The transcriptomic analysis of Cookson et al. [102] suggested that sucrose affects gene expression via multiple routes at the transcription level. One of the transcriptional responses to changes in carbon status is the signaling via SnRK1 accounts. The I-box (light regulated) and ABRE-like motifs are enriched in all the clusters of gene induced by carbon depletion, and promoters of *BnCIPK9* and *AtCIPK9* also carry several I-box elements.

Previous studies indicated that CIPK15 induced the accumulation of SnRK1A, which promotes the interaction between MYBS1 and the TA box and regulates the transcription level of *α*-*Amy3* [103, 104]. In the beginning of germination, signals of nutrient starvation induce the nuclear import and expression of MYBS1, which activates target gene expression by binding to the TA box in the promoters of the target gene. In rice, during early postgermination growth, MYBS1 plays an important role in the common nutrient-starvation signaling pathway, possibly through CBL–CIPK15–SnRK1A-dependent sugar-starvation signaling pathway [30, 103, 104]. Moreover, SnRK1 has been reported to play a pivotal role in linking stress, development, and sugar signaling at the level of gene expression, which indicates its crucial regulatory effect on plant metabolism, energy balance, and growth [24]. These findings suggest that function conservation in SnRK1/SNF1/AMPK has played an important role in sugar-mediated regulation across eukaryotes throughout evolution. Consistent with the results obtained for rice, the promoter region of *BnCIPK9* would allow the identification of *cis*-acting elements and *trans*-acting factors involved in sugar and lipid accumulation in rapeseed.

## Conclusion

Overall, this study shows that *BnCIPK9* and *AtCIPK9* encode a protein kinase involved in sugar-related response and are important regulatory elements in energy storage. Gene *AtCIPK9* has a significant effect on early seedling root establishment in *A. thaliana*. Although it has been confirmed that sugars suppress the repression of *BnCIPK9* and *AtCIPK9*, it is necessary, and would be interesting, to explore the roles of *BnCIPK9* and *AtCIPK9* in the sugar signaling pathway and to identify the downstream targets of CIPK9 in plants. In the future, genomewide transcriptomic and proteomic analyses combined with in vitro and in vivo studies of protein–protein interaction should provide more information on the function of CIPK9 in the sugar response.

## Methods

### Plants material and growth condition

Rapeseed parent plants with high-oil (P1) and low-oil (P2) contents, and the F_2_ populations resulting from them were grown in a randomized array in Saskatoon, Canada. The dataset consists of seven lines of high OC (H4, H12, H41, H64, H86, H133, H154) and their pool (HP), seven lines of low OC (L89, L161, L179, L267, L270, L306, L307) and their pool (LP), and hybrids of the high- and low-oil lines (P1P2, P2P1), all belonging to F_2_ populations. The *B. napus* transgenic lines used for the analysis of OC were grown during the normal growing season at the experimental station of Huazhong Agricultural University, China. Regular field management was conducted according to local agricultural practices.

*Arabidopsis thaliana* ecotype Columbia (Col-*0*) was grown at 22 °C in growth chambers under 16 h of light and 8 h of darkness. *A. thaliana* Col-*0* and its *cipk9*, *cbl2*, and *cbl3* mutants were ordered from the Arabidopsis Biological Resource Center (ABRC), USA (http://www.arabidopsis.org/). Genomic DNA was extracted from plants grown in soil for 1 month. Homozygous lines of *AtCIPK9* (SALK_058629), *AtCBL2* (SALK_057048C), and *AtCBL3* (SALK_091827C) mutants were screened by PCR using LBb1.3 and three gene-specific primers (Additional file 1: Table S1). We chose etiolated seedlings grown on half-strength MS medium (Caisson Laboratories Inc., UT, USA) without supplementary sugar as a model system for carbon starvation. The *A. thaliana* seeds (Col-*0*, *cipk9*, com-1, com-2, *cbl2*, *cbl3*) were surface sterilized, and then randomly sown on half-strength MS agarose plates with 1% sucrose, or without sugar.

### Construction of plasmids

All primers for cloning and vector construction are listed in Additional file 1: Table S1. Both complementation lines of *cipk9*, with the *AtCIPK9* genomic sequence (com-1:promoter*AtCIPK9*:gDNA(*AtCIPK9*)) and coding sequence (com-2:promoter*AtCIPK9*:CDS(*AtCIPK9*)) driven by its own promoter, were cloned into the PCAMBIA1300 vector, and were transformed into the *cipk9* (SALK_058629) mutant by floral dipping. Transgenic lines were selected on half-strength MS medium containing 1% agarose and supplemented with 40 µg mL^−1^ hygromycin, and T3 seedlings were planted on vertical half-strength MS agarose plates with or without 1% sucrose for 7 days. The deletion of the NAF motif and substitution of threonine with aspartate within the activation loop of *BnCIPK9* were performed via gene splicing by overlap extension PCR in vitro mutagenesis. To obtain *BnCIPK9*-overexpression transgenic lines, the kinase domain of CIPK9T178D was cloned a pC2300 vector harboring *BnNapin* promoter and a *CaMV35* *s* polyA addition sequence, and transformed into rapeseed variety J572 (wild type). The rapeseed transgenic lines were generated using *A. tumefaciens*-mediated transformation [105, 106]. Primers used in the positive transplant test are listed in Additional file 1: Table S1. The four independent transgenic lines mentioned above (at least eight plants per line) were selected in the T2 generation and used in the current study.

### Construction of promoter:GUS transgenic plants

The *BnCIPK9* promoter1 (3050 bp) and *BnCIPK9* promoter2 (3372 bp) fragments were amplified with a forward primer containing a *Sal*I restriction site and a reverse primer containing a *Bam*HI restriction site, and then cloned into the pCXGUS-P vector in front of the GUS coding sequence. The *AtCIPK9* promoter fragment (3000 bp) was amplified and cloned into pCXGUS-P in front of the GUS coding sequence using *Sal*I and *Bam*HI sites. Multiple lines of *BnCIPK9* promoter1:GUS, *BnCIPK9* promoter2:GUS, and *AtCIPK9* promoter:GUS seedlings were generated, and the homozygous lines were isolated and used to verify the reproducibility of GUS staining patterns. The seeds of T3 transgenic lines (*BnCIPK9*promoter1:GUS, *BnCIPK9* promoter2:GUS and *AtCIPK9* promoter:GUS) were surface-sterilized, and then randomly sown on half-strength MS agarose plates with 1% sucrose, 3% sucrose, 1% glucose, 3% glucose, 1% mannitol (Sigma-Aldrich, MO, USA), 3% mannitol or without sugar supplementation. The plates were incubated for 1 week (22 °C/18 °C; 16/8 h day/night photoperiod). Analysis of GUS activity in different tissues of T3 transgenic lines was performed as described previously [107].

### Quantification of GUS activity

The expression pattern of the promoters was determined by a quantitative GUS activity assay using the total protein extracted from plants grown for 7 days on half-strength MS without sugar supplementation or with 1% sucrose, 3% sucrose, 1% glucose, 3% glucose, 1% mannitol, or with 3% mannitol. Fluorometric GUS assays to measure GUS activity in plant seedlings were performed according to Jefferson [107]. The total protein concentration in plant extracts was determined according to Bradford [108] using bovine serum albumin as the standard. Fluorescence was recorded using Tecan Infinite M200 PRO (Tecan Group Ltd., Switzerland) [109].

### Fatty acid and lipid analysis in *A. thaliana*

Both fatty acid composition and lipid content of Col-*0*, *cipk9*, com-1, and com-2 plants were analyzed by gas chromatography (GC) following previously published procedures [6]. About 100 dry seeds of each background were weighted for the first biological repeat, and at least three technical repeats were included in each experiment. The samples were transmethylated at 90 °C for 60 min, and 200 µg of heptadecanoic acid (C17:0) was used as the quantitative internal standard. After cooling the tubes to room temperature, 1.5 mLof 0.9% NaCl (w/v) was added to the mix, and the FA methyl esters (FAMEs) were extracted twice in 1 mL of hexane. Samples were then analyzed with GC using a flame ionization detector (FID) on Agilent 6890 (Agilent, santa Clara, CA, USA), employing helium as the carrier gas.

### Analysis of OC in rapeseed

The total OC in rapeseed seeds was measured in Foss NIR-System 5000 near-infrared reflectance spectroscope (NIR-Systems, Inc., Silver Spring, MD, USA) [110], using the parameters described by Gan et al. [111]. At least eight biological replicates from each transgenic line were used.

### RNA isolation and quantitative real-time PCR

Total RNA was isolated from the plant samples (root, stem, flower, bud, 24DAP seed, and 24DAP silique wall of the parent P1) and used for quantitative real-time PCR (qRT-PCR). The RNA contained in each sample was quantified in a NanoDrop2000 and its integrity was checked on 1.2% (w/v) agarose gels. The qRT-PCR assay was performed on a CFX96 real-time PCR machine (Bio-Rad, USA) using gene-specific primers and the SYBR Green PCR Master Mix (Applied Biosystems), according to the manufacturer’s instructions. For the internal control, we used species-specific *actin* primer sets for *B. napus* and *A. thaliana*. All qRT-PCR experiments were performed in triplicate for each sample from three independent biological replicates. All primers for the qRT-PCR are listed in the Additional file 1: Table S1.

### Statistical analysis

To ensure reproducibility, the experiments were performed using at least three biological triplicates. All quantitative data were expressed as the mean value with corresponding standard deviation (SD), and statistical differences between means was evaluated using the Student’s *t* test, at significance levels of *P *< 0.05 and *P *< 0.01.

## Additional file

**Additional file 1: Table S1.** Primers used in the present study.

## Abbreviations

ACC1: acetyl CoA carboxylase
AMPK: AMP protein kinase
bp: base pairs
CBL: calcineurin B-like protein
CIPK: CBL-interacting protein kinase
TAG: triacylglycerol
DAP: day after pollination
FA: fatty acid
GC: gas chromatography
MS: Murashige and Skoog
OC: seed oil content
PCR: polymerase chain reaction
SNF1: sucrose non-fermenting
SnRK: sucrose non-fermenting kinase
